# Decease of Prof. John Butterfield

**Published:** 1849-11

**Authors:** 


*Decease of Prof. John Butterfield.*—We had just written the foregoing notice, which was excluded from our last number for want of space, when we saw, in one of our contemporaries, the announcement of the decease of Prof. John Butterfield, of the Starling Medical College, in Columbus, Ohio, and editor of the Ohio Medical and Surgical Journal. Prof B. possessed talents and acquirements eminently fitting him for the laborious and responsible duti.s of teacher and editor. He has been cut down in the prime of life, when all that should have satisfied human aspirations, in this existence, appeared opening before him. The medical profession has lost a highly gifted and useful member; but we trust he is now enjoying the fruition of hopes not restricted to objects of earth.

Phthisis, the disease of which he died, had long been preying on his system. In the last number of his excellent Journal, he says, "many of our articles, like the present one, have been written while suffering under bodily pain and infirmity. We have hardly been able to do justice to ourselves, but we hope all the while for better times." His hopes, alas! were doomed to disappointment, but his friends may console themselves with the reflection, that, although to labor usefully in this world is to do well, to enjoy the rewards of useful labors in another world, is far better.

